# SIRT1 deacetylase in aging‐induced neuromuscular degeneration and amyotrophic lateral sclerosis

**DOI:** 10.1111/acel.12839

**Published:** 2018-10-08

**Authors:** Adrianna Z. Herskovits, Tegan A. Hunter, Nicholas Maxwell, Katherine Pereira, Charles A. Whittaker, Gregorio Valdez, Leonard P. Guarente

**Affiliations:** ^1^ Department of Pathology Beth Israel Deaconess Medical Center, Harvard Medical School Boston Massachusetts; ^2^ Department of Biology Massachusetts Institute of Technology Cambridge Massachusetts; ^3^ University of Miami Miller School of Medicine Miami Florida; ^4^ Virginia Tech Carillion Research Institute Virginia Tech Roanoke Virginia; ^5^ Department of Biological Sciences Virginia Tech Blacksburg Virginia

**Keywords:** aging, amyotrophic lateral sclerosis, NAD, neurodegenerative disease, neuromuscular junction, SIRT1

## Abstract

SIRT1 is an NAD^+^‐dependent deacetylase that functions in a variety of cells and tissues to mitigate age‐associated diseases. However, it remains unknown if SIRT1 also acts to prevent pathological changes that accrue in motor neurons during aging and amyotrophic lateral sclerosis (ALS). In this study, we show that SIRT1 expression decreases in the spinal cord of wild‐type mice during normal aging. Using mouse models either overexpressing or lacking SIRT1 in motor neurons, we found that SIRT1 slows age‐related degeneration of motor neurons’ presynaptic sites at neuromuscular junctions (NMJs). Transcriptional analysis of spinal cord shows an overlap of greater than 90% when comparing alterations during normal aging with changes during ALS, revealing a substantial upregulation in immune and inflammatory response genes and a downregulation of synaptic transcripts. In addition, overexpressing SIRT1 in motor neurons delays progression to end‐stage disease in high copy SOD1^G93A^ mice. Thus, our findings suggest that there are parallels between ALS and aging, and interventions to impede aging may also slow the progression of this devastating disease.

## 1 INTRODUCTION

SIRT1 plays a critical role in mitigating damages caused by aging and a number of neurodegenerative diseases including amyotrophic lateral sclerosis (ALS) (Kim et al., 2007; Watanabe et al., 2014). SIRT1 is a nicotinamide adenine dinucleotide (NAD+)‐dependent deacetylase that catalyzes the removal of acetyl groups from lysine residues on histone proteins, resulting in gene silencing (Braunstein, Rose, Holmes, Allis, & Broach, 1993; Imai, Armstrong, Kaeberlein, & Guarente, 2000; Tanny, Dowd, Huang, Hilz, & Moazed, 1999). SIRT1 also deacetylates transcription factors including PGC1α, which induces mitochondrial oxidative phosphorylation (Lagouge et al., 2006), p53, which promotes cell survival (Luo et al., 2001; Vaziri et al., 2001) and triggers adaptation to calorie restriction with its accompanying stress resistance (Guarente, 2013). Through these actions, SIRT1 functions to maintain cellular homeostasis and thereby prevent age‐related pathological changes. Unfortunately, the activity of SIRT1 decreases with advancing age in many cells and tissues either due to a decrease in protein levels (Gong et al., 2014; Sakamoto, Miura, Shimamoto, & Horio, 2004), a reduction in NAD+levels (Imai & Guarente, 2014) or both.

In addition to modulating transcription factors and metabolic enzymes, SIRT1 has been shown to directly deacetylate proteins that impact neurodegenerative disorders (Herskovits & Guarente, 2014; Min et al., 2010; Montie, Pestell, & Merry, 2011). Studies using resveratrol, a polyphenolic compound that activates SIRT1 and other targets, have shown mixed effects in the SOD1^G93A^ mouse model of ALS, with some dosing regimens showing protection and others indicating no effect on disease progression (Han, Choi, Soon Shin, & Kang, 2012; Kim et al., 2007; Mancuso et al., 2014; Markert, Kim, Gifondorwa, Childers, & Milligan, 2010; Song, Chen, & Zhang, 2014). Overexpressing PGC1α slows ALS‐related pathologies in SOD1^G93A^ mice (Zhao et al., 2011), and more directly, elevating SIRT1 expression using the prion promoter has shown lifespan extension in the SOD1^G93A^ low copy transgenic mouse line (Watanabe et al., 2014). However, these findings do not address the focal site of action for SIRT1 or explain why an antiaging protein would impact ALS.

A shared feature of normal aging and ALS is the progressive degeneration of the neuromuscular junction (NMJ), a synapse that is essential for the function of alpha‐motor neurons and skeletal muscles (Valdez, Tapia, Lichtman, Fox, & Sanes, 2012). With advancing age or ALS, the NMJ acquires deleterious structural and functional features that include the loss of synaptic vesicles and dysregulated neurotransmitter release that lead to the degeneration of motor axon nerve endings (Jang & Van Remmen, 2011; Valdez et al., 2010; Arbour, 2015). To date, it has been postulated that oxidative stress, mitochondrial dysfunction, impaired autophagy, and decreased release of trophic factors (Carnio et al., 2014; Park, 2015) contribute to the degeneration of NMJs with advancing age and may also affect ALS. SIRT1 has been shown to influence stress resistance, mitochondrial oxidative phosphorylation, and autophagy (Lagouge et al., 2006; Lee et al., 2008), suggesting that this protein could also impact NMJ degeneration during normal aging and neuromuscular disease. Supporting this possibility, resveratrol was recently shown to slow age‐induced degeneration of NMJs in mice (Stockinger, Maxwell, Shapiro, deCabo, & Valdez, 2017).

In the current study, we examined the effect of SIRT1 overexpression and depletion on NMJs during aging and progression of ALS using the SOD1^G93A^ mouse model. We discovered that increasing SIRT1 in motor neurons partially protects NMJs from the deleterious effects of aging and ALS. Moreover, we uncovered a surprising degree of transcriptional overlap between aged and ALS‐affected spinal cords. These findings suggest that SIRT1 may be a target for slowing degeneration of NMJs, and the resulting loss of motor function, that occurs in ALS and during aging.

## 2 RESULTS

### 2.1 SIRT1 during normal aging and neuromuscular disease

**Figure 1:**
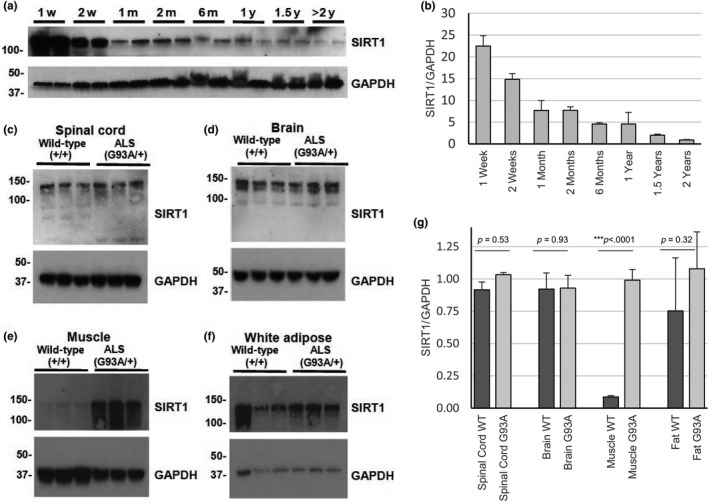
SIRT1 levels in mouse spinal cord during normal aging and neuromuscular disease. (a) Western blot of spinal cord homogenate from postnatal C57Bl6 mice ranging in age from one week to over two years. One female mouse and one male mouse were assayed at each timepoint. (b) Densitometry was performed to compare SIRT1 levels after normalization to GAPDH at different ages. (c–f) Immunoblots of central nervous system and metabolic tissues from three male SOD1^G93A^ mice and three litter‐ and gender‐matched wild‐type mice were analyzed at 5.5 months of age. (g) Densitometry was performed to compare SIRT1 levels after normalization to GAPDH between wild‐type and SOD1^G93A^ mice. Unpaired *t* ‐tests were used to compare levels of SIRT1 normalized to GAPDH in wild‐type and SOD1^G93A^ animals for each tissue analyzed. Values reported as mean ± *SD*.

In order to understand whether SIRT1 might play an important role in motor function during aging, we examined SIRT1 levels in spinal cords from C57Bl/6 mice at multiple time points. This analysis revealed that SIRT1 decreases sharply from birth to young adulthood, and levels continue to decline as mice transition into old age (Figure 1a,b). These findings are consistent with observations that SIRT1 declines in brain, liver, muscle, heart, and adipose tissue during aging (Gong et al., 2014; Sakamoto et al., 2004). These results raise the possibility that SIRT1 may be similarly dysregulated in ALS, a disease that alters fat metabolism and causes muscle wasting and motor neuron loss. We surveyed SIRT1 levels in the central nervous system, skeletal muscles and other tissues from SOD1^G93A^ mice at approximately 5.5 months of age. We did not observe significant alterations in levels of SIRT1 in spinal cord, brain, and adipose tissue, but found a substantial upregulation in skeletal muscle of SOD1^G93A^ mice relative to control mice (Figure 1c–g). Although lower motor neurons in the spinal cord are a major focus in ALS, these findings suggest that SIRT1 may be dysregulated in skeletal muscle during the disease process.

Among spinal cord cells, we hypothesized that loss of SIRT1 expression and function might be particularly detrimental to motor neurons because transport via long motor axons is an energetically demanding process. To assess the effect of SIRT1 on motor neurons, we examined several previously described mouse lines that can be engineered to overexpress or inactivate SIRT1 in motor neurons (Cohen, Supinski, Bonkowski, Donmez, & Guarente, 2009; Firestein et al., 2008; Rossi et al., 2011). Transgenic mice were created by breeding animals with a floxed‐stop cassette upstream of *Sirt1* with mice expressing Cre recombinase under the choline acetyltransferase promoter. Tissue‐specific knockout mice were created by breeding animals with loxP sites flanking exon 4 of *Sirt1* with animals expressing Cre recombinase under the choline acetyltransferase promoter to excise the catalytic domain. We analyzed motor axons presynaptic sites at NMJs because they degenerate before atrophic changes are apparent in the soma and along axons. NMJs were examined in whole‐mounted extensor digitorum longus (EDL) muscles by labeling the presynaptic region of motor axons with an antibody against synaptotagmin‐2 (Syt2), a protein that associates with synaptic vesicles, and marking the postsynaptic region with fluorescently tagged alpha‐bungarotoxin, which binds with high affinity to muscle nicotinic acetylcholine receptors (AChRs). Innervation was scored based on the overlap between pre‐ and postsynaptic regions.

**Figure 2:**
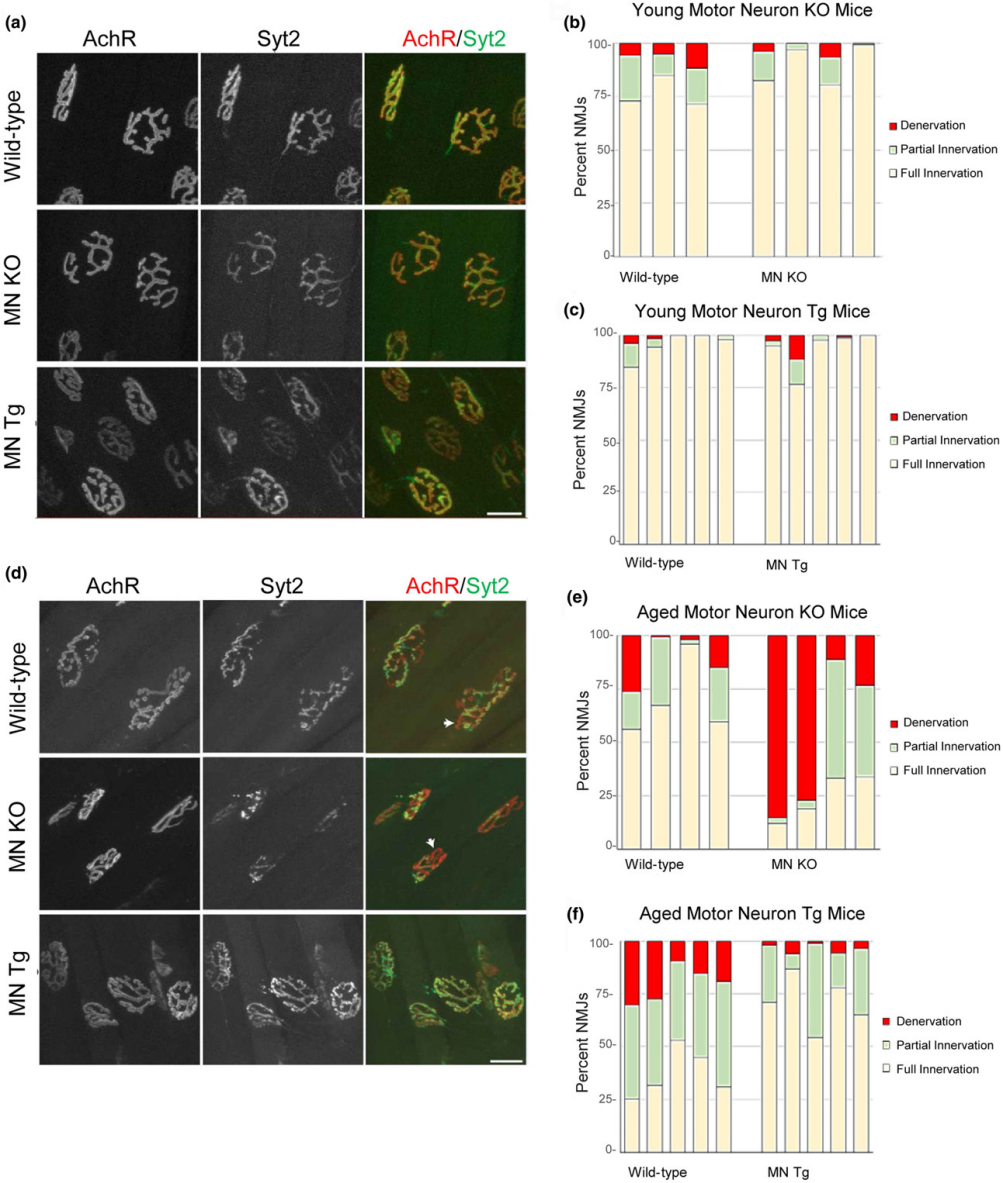
SIRT1 protects the neuromuscular junction during normal aging. (a) Representative images of motor axons labeled with synaptotagmin 2 (Syt2; Green) and nicotinic acetylcholine receptors (AChR; Red) marked with α‐bungarotoxin at NMJs from young mice analyzed at 3–4 months of age. (b) Full innervation, partial innervation, and denervation of neuromuscular junctions from four male motor neuron knockout mice and three litter‐ and gender‐matched control mice at 3–4 months of age. (c) Full innervation, partial innervation, and denervation of neuromuscular junctions from five male motor neuron transgenic mice and five litter‐ and gender‐matched control mice at 3–4 months of age. (d) Representative images of motor axons labeled with synaptotagmin 2 (Syt2; Green) and nicotinic acetylcholine receptors (AChR; Red) marked with α‐bungarotoxin at NMJs from aged mice analyzed at 18–24 months of age. White arrows indicate partially innervated NMJs. Scale bar = 20 μm. (e) Full innervation, partial innervation, and denervation of neuromuscular junctions from four male motor neuron knockout mice and four litter‐ and gender‐matched control mice at 18–24 months (average: 21.2 months, range: 19–24 months) of age. (f) Full innervation, partial innervation, and denervation of neuromuscular junctions from five male motor neuron transgenic mice and five litter‐ and gender‐matched control mice at 18–24 months (average: 21.9 months, range 21–24 months) of age.

We found that SIRT1 is dispensable for the normal development and stability of NMJs in young animals. In mice at 3–4 months, NMJs were indistinguishable between control, SIRT1 overexpressing, and knockout mice (Figure 2a–c). We next examined NMJs in wild‐type, SIRT1 transgenic mice and knockout animals at 18–24 months (Figure 2d). We noted that wild‐type mice showed partial denervation of NMJs at this age, as expected due to the normal aging process (Valdez et al., 2010). SIRT1 deletion in motor neurons exacerbated this decline with knockout animals exhibiting less NMJ innervation at older ages (Figure 2e, Supporting Information Figure S1), and SIRT1 overexpression suppressed the decline compared to wild‐type littermate controls with transgenic animals showing more NMJ innervation at 18–24 months (Figure 2f, Supporting Information Figure S1). These findings demonstrate that SIRT1 protects NMJs from the damaging effects of aging.

Since aging and ALS have similar effects on motor neurons presynaptic sites at NMJs, we carried out RNA‐seq to gain insight into transcriptional changes that occur in the spinal cord during aging and ALS pathogenesis in SOD1^G93A^ mice. We focused on differentially expressed protein‐coding genes defined as those having an absolute log fold change greater than 1 and adjusted p‐value less than 0.05 (Supporting Information Table S1). Mouse genes were mapped to human orthologs using Mouse Genome Informatics orthology reports, and pathway analysis was performed based on Gene Set Enrichment Analysis (GSEA) (Mootha et al., 2003).

**Figure 3:**
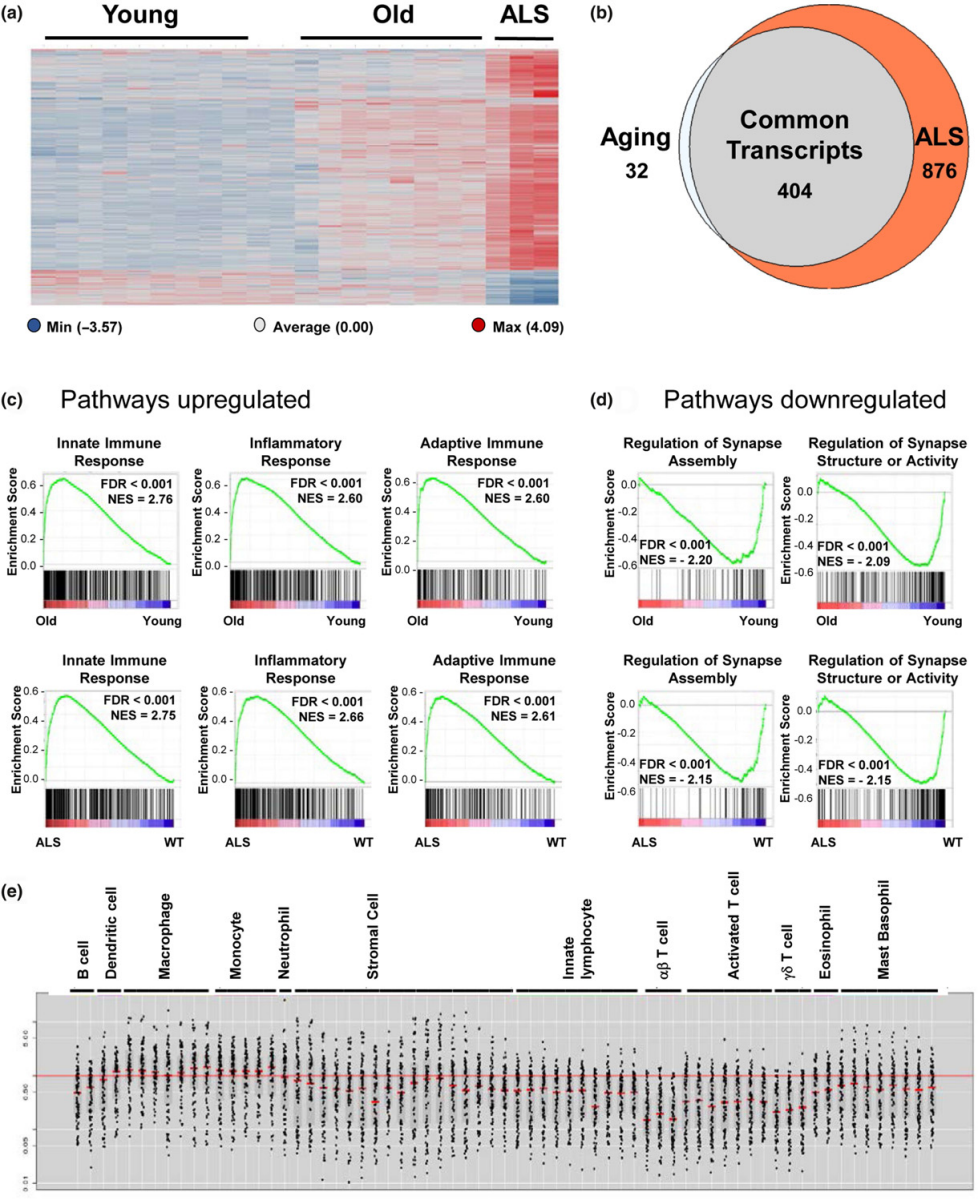
Similar transcriptional alterations occur during aging and amyotrophic lateral sclerosis. (a) Heatmap comparing differentially expressed protein‐coding transcripts (absolute log fold change >1 and adjusted *p*‐value <0.05) in whole spinal cord from young wild‐type compared with old wild‐type and symptomatic SOD1^G93A^ mice compared with wild‐type controls. Dataset includes eight young male mice at 3–4 months and eight old male mice at 18–24 months to examine aging‐related changes. Whole spinal cords from three SOD1^G93A^ mice (two female and one male) and three wild‐type control mice (two female and one male) at 5 months were used to examine changes due to ALS. Red signal indicates high relative expression, and blue signal indicates low relative expression. (b) Venn diagram illustrating the overlap between transcripts upregulated (log fold change >1 and adjusted *p*‐value <0.05) in aged spinal cords compared with young controls and ALS spinal cords compared with healthy controls. (c) Major representative pathways upregulated in aging and disease cohorts. (d) Major representative pathways downregulated in aging and disease cohorts. (e) Upregulated genes in aging and ALS with highest fold change are compared with ImmGen transcriptional profiles for major classes of immune cells. The means‐normalized expression value of each gene in different immune cell populations is shown as a scatter plot.

Supervised clustering of these genes revealed a remarkable overlap between aging and ALS (Figure 3a). We found that over 90% of transcripts upregulated in aged spinal cords (representing the vast majority of age‐sensitive transcripts) were also elevated in ALS relative to their respective control cohorts (Figure 3b). GSEA analysis of the 404 transcripts that overlap indicates these genes largely mediate inflammatory, innate, and adaptive immune processes (Figure 3c). Analysis of downregulated transcripts in common between aged and ALS‐affected spinal cords revealed genes known to mediate the assembly, stability, and function of synapses (Figure 3d).

Transcripts with the highest level of expression increased in both ALS and aging were compared with data generated by the Immunological Genome (ImmGen) project, a consortium that has mapped gene expression patterns in major classes of immune cell types (Heng & Painter, 2008). The purpose of this analysis was to understand the cellular basis underlying the increased immune and inflammatory response during aging and ALS. Transcripts upregulated in both the ALS vs. wild‐type and the old vs. young comparisons were compared with transcriptional profiles of immune cell populations in the ImmGen v2 dataset to generate a W‐plot (Figure 3e). In this analysis, the y‐axis is the ratio of gene expression value in one population relative to the average in all populations and the red line at *y* = 1 indicates the point where the gene expression value in a given immune cell population is equal to the average value in all populations. The vertical columns represent different immune cell populations, and some cell types have more representation within this ImmGen database relative to other classes of immune cells. The major populations increased in ALS and aging were from monocyte and macrophage lineages. These changes are consistent with other studies that have found robust activation of these cells as part of the immune response during ALS disease progression and aging (Butovsky et al., 2012; Chiu et al., 2009; Galbavy et al., 2017; Kullberg, Aldskogius, & Ulfhake, 2001; Zondler et al., 2016).

**Figure 4:**
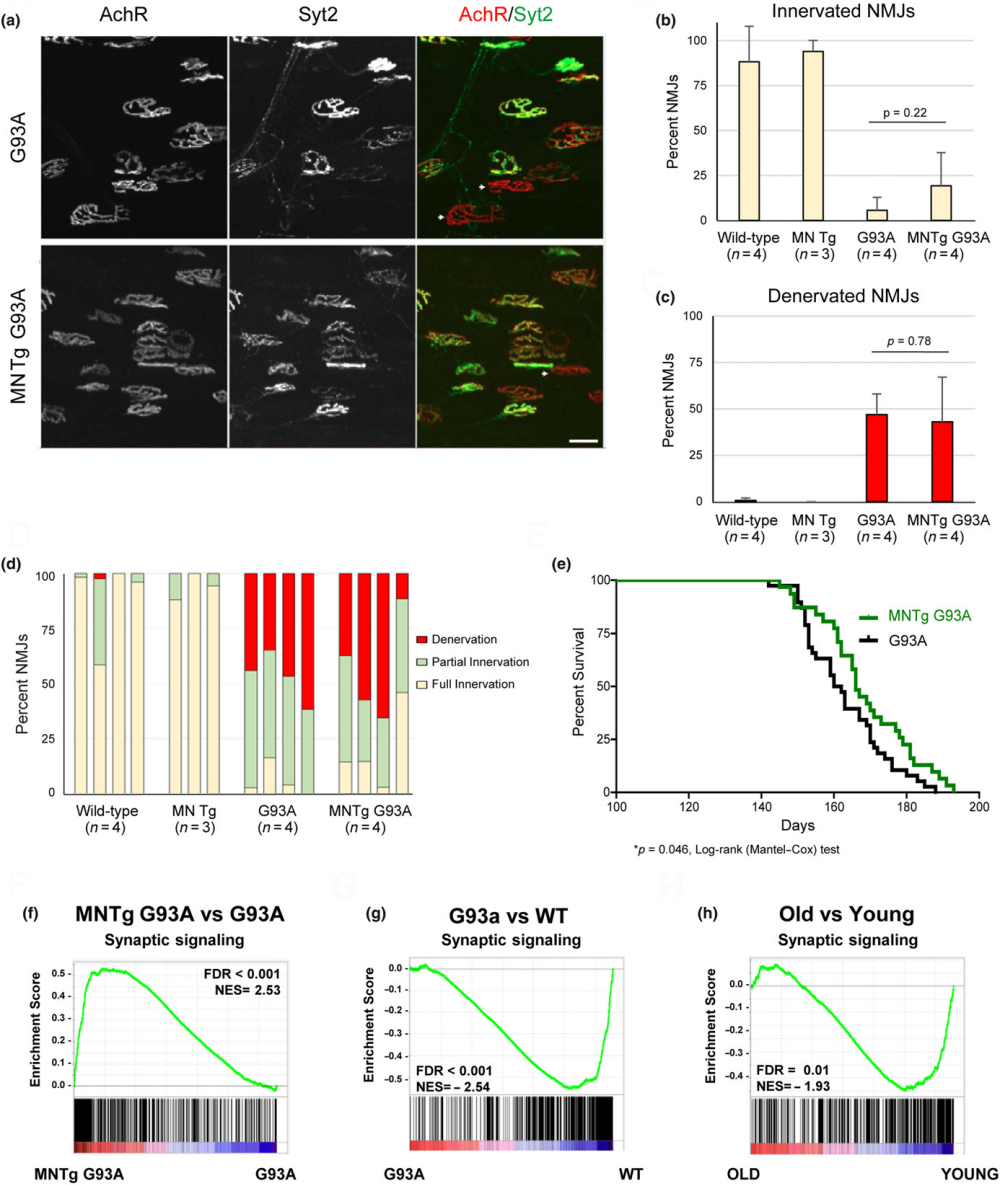
SIRT1 overexpression in motor neurons is protective in the SOD G93A model of amyotrophic lateral sclerosis. (a) Representative images of NMJ clusters in symptomatic SOD1^G93A^ and SOD1^G93A^ mice that overexpress SIRT1 in motor neurons. White arrows point to denervated NMJs. Scale bar = 20 μm. (b) Quantification of innervated NMJs from wild‐type (four male), motor neuron transgenic (three male), SOD1^G93A^ (three male, one female), and SOD1^G93A^ mice that overexpress SIRT1 (three male, one female). Unpaired *t* tests were used to compare innervation in litter‐matched SOD1^G93A^ and SOD1^G93A^ mice that overexpress SIRT1. Values reported as mean ± *SD*. (c) Quantification of denervated NMJs from wild‐type (four male), motor neuron transgenic (three male), SOD1^G93A^ (three male, one female), and SOD1^G93A^ mice (three male, one female) that overexpress SIRT1. Unpaired *t*‐tests were used to compare denervation in litter‐matched SOD1^G93A^ and SOD1^G93A^ mice that overexpress SIRT1. Values reported as mean ± *SD*. (d) NMJs were characterized as innervated, partially innervated, or denervated depending on the degree of overlay between the motor axon and AChR clusters and quantification of NMJ innervation. (e) Kaplan–Meier survival curve comparing SOD1^G93A^ with litter‐ and gender‐matched motor neuron transgenic SOD1^G93A^ mice that overexpress SIRT1 in motor neurons (16 male SOD1^G93A^ mice; 15 male MNTg SOD1^G93A^; 22 female SOD1^G93A^ mice; 16 female MNTg SOD1^G93A^ mice). *p < 0.05, log‐rank (Mantel–Cox) test. Comparison of synaptic signaling pathways in (f) motor neuron transgenic SOD1^G93A^ relative to SOD1^G93A^ mice (g) SOD1^G93A^ relative to wild‐type mice and (h) old relative to young mice.

To further explore our findings that genes known to mediate the function of synapses are decreased in both aging and ALS, we tested the impact of SIRT1 overexpression in motor neurons in the SOD1^G93A^ line. We did not observe a statistically significant difference in the number of innervated or denervated NMJs in a cohort of three to five sib pair controlled ALS mice overexpressing SIRT1 in motor neurons (Figure 4a–d). Nonetheless, overexpression of SIRT1 in motor neurons delayed disease progression such that transgenic mice reached disease endpoint five days later than gender‐ and litter‐matched sib pairs with wild‐type levels of SIRT1 (Figure 4e).

To investigate whether SIRT1 influences synaptic signaling pathways in ALS motor neurons, we used transcriptomic analysis to compare MNTg SOD1^G93A^ mice with SOD1^G93A^ sib pairs. Using GSEA tools to detect coordinated changes in functionally related genes, we observed a statistically significant enrichment in synaptic signaling (FDR <0.001, NES = 2.53) in MNTg SOD1^G93A^ mice relative to sib pair controls overexpressing SOD1^G93A^. We found that this gene set is decreased both during aging of wild‐type animals and during ALS progression and these changes are reversed in the MNTg G93A mice (Figure 4f–h). These findings demonstrate that SIRT1 plays an important role in motor neurons, preserving the integrity of motor units during aging and ALS disease progression.

Another interesting set of genes reduced in SOD1^G93A^ animals relative to wild‐type mice and increased in MNTg SOD1^G93A^ relative to SOD1^G93A^ are oxidative phosphorylation and mitochondrial respiratory chain I biogenesis genes (Supporting Information Figure S2). Several targets of SIRT1 including PGC‐1α and FoxO1/3 have been previously shown to influence mitochondrial biogenesis, antioxidant formation, and inflammation (Brunet, 2004; Lagouge et al., 2006). While we did not observe a statistically significant impact on these pathways in wild‐type mice with aging (data not shown), it is possible that an effect might have been apparent in older animals, as the SOD1^G93A^ exhibit more severe neuromuscular pathology that is observed during normal aging.

## 3 DISCUSSION

The goal of this study was to examine the impact of SIRT1 on NMJs during aging and affected by ALS using the SOD1^G93A^ mouse model. We found that SIRT1 is dispensable for the normal development and stability of NMJs in young mice; however, SIRT1 overexpression helped protect motor neurons from the damaging effects of aging in older animals. SIRT1 overexpression slowed disease progression in the high copy SOD1^G93A^ model of ALS, and transcriptional analysis of spinal cord showed extensive overlap in transcriptional changes that occur centrally in the SOD1^G93A^ model of ALS and during normal aging of wild‐type mice with a substantial upregulation of immune and inflammatory response genes.

In presymptomatic SOD1^G93A^ mice, immune activation in the peripheral and central nervous system begins focally and becomes widespread after clinical onset (Alexianu, Kozovska, & Appel, 2001; Chiu et al., 2009; Hall, Oostveen, & Gurney, 1998). Macrophage activation in the peripheral nervous system occurs in parallel with microglial activation and T‐cell infiltration in the spinal cord (Chiu et al., 2009). During the disease process, spinal cord‐derived microglia recruit inflammatory monocytes, and this process correlates with neuronal loss (Butovsky et al., 2012). During aging, immune cell infiltration also begins long before late‐stage degeneration with increases in T cell, microglial, and macrophage markers seen in the spinal cord during middle age (Galbavy et al., 2017; Galbavy, Kaczocha, Puopolo, Liu, & Rebecchi, 2015; Kullberg et al., 2001). One of the molecular mechanisms underlying this change may be the deacetylation of IFN regulatory factor 8 (IRF8) by SIRT1, which negatively regulates inflammatory cytokine production and affects macrophage infiltration during inflammation (Jia et al., 2017). Many degenerative and inflammatory changes have been previously reported in both ALS and aging; however, our study is the first to report the extensive nature of transcriptional overlap in these conditions.

In contrast to the many proinflammatory genes that were upregulated, we identified fewer downregulated transcriptional pathways in ALS and aging. However, one of the patterns that emerged was a general loss of transcripts associated with synapse activity and structure, consistent with the structural and functional degeneration we noted at the neuromuscular synapse by morphological analysis in the aging and ALS‐affected cohorts. The reversal of these transcriptional changes in MNTg SOD1^G93A^ mice relative to SOD1^G93A^ animals supports the hypothesis that SIRT1 plays an important role in preserving synaptic function.

Our transcriptional analysis also showed that SIRT1 overexpression in motor neurons suppresses changes in transcripts involved in oxidative phosphorylation and mitochondrial respiratory chain complex 1 biogenesis in SOD1^G93A^ mice (Supporting Information Figure S2). Several targets of SIRT1 including PGC‐1α and FoxO1/3 have been previously shown to influence mitochondrial biogenesis, antioxidant formation, and inflammation (Brunet, 2004; Lagouge et al., 2006). These findings are interesting because mitochondrial dysfunction and the formation of reactive oxygen species are thought to occur in SOD1 mutation carriers as well as patients with C9ORF72 mutations, mutant TDP43, and FUS during ALS pathogenesis (Lopez‐Gonzalez et al., 2016; Onesto et al., 2016).

One of the important future directions of this work will be to investigate whether SIRT1 activity in motor neurons is altered during ALS pathogenesis. One approach to this experiment is to investigate the acetylation status of well‐characterized SIRT1 targets. However, many of the acetyl‐specific antibodies that are reliable in cell culture are not sufficiently sensitive to detect post‐translationally modified proteins expressed endogenously in animal tissues. Moreover, motor neurons comprise only 2–3 percent of total spinal cord input, and isolating these cells from an adult animal is technically difficult. Another confound is that SIRT1 substrates are targets for multiple histone deacetylases, so these experiments will require highly sensitive and specific techniques.

Identifying the tissue type responsible for a disease‐modifying intervention is important because penetration of bioactive compounds must occur in the appropriate compartment for a therapy to be effective. In our study, we did not observe significant alteration in protein levels of SIRT1 in either brain or spinal cord in late‐stage SOD1^G93A^ mice. However, we observed a substantial increase in SIRT1 in skeletal muscle from ALS mice (Figure 1e), possibly a consequence of denervation and fiber type switching that occurs during the disease process. We found that overexpressing SIRT1 in skeletal muscles does not affect disease progression (data not shown) and also observed that deleting SIRT1 in skeletal muscles from SOD1^G93A^ mice has no effect on the innervation status of NMJs (Supporting Information Figure S3). These findings suggest that SIRT1 in skeletal muscles does not play a crucial role in NMJ degeneration and ALS progression.

While this mouse model does recapitulate many features of ALS, mice do not spontaneously develop motor neuron disease when endogenous SOD1 is mutated. Moreover, the majority of patients with ALS have sporadic disease and do not harbor the SOD1^G93A^ mutation. Human studies are more likely to be carried out using pharmacotherapies initiated after patients become symptomatic and these treatments are also subject to metabolism and off‐target effects.

Our findings raise the possibility that interventions aimed at slowing aging might also slow the progression of ALS. In particular, SIRT1 activator compounds or NAD^+^ precursors may be of benefit, but any efficacy will demand that compounds cross the blood–brain barrier to reach motor neurons. Drugs that modulate inflammation by targeting macrophage activation and monocyte recruitment may be another promising approach. In conclusion, our findings suggest that ALS may be considered an extreme form of spinal cord aging that targets motor neurons, and that strategies to slow aging may be effective treatments for this disease.

## 4 EXPERIMENTAL PROCEDURES

### 4.1 Mouse studies

All animal experiments were performed in accordance with protocols approved by the Institutional Animal Care and Use Committee of Massachusetts Institute of Technology. Motor neuron transgenic mice were created by breeding animals with a floxed‐stop cassette upstream of mouse Sirt1 cDNA in the collagen A1 locus (received from Sinclair Lab, also available from Jackson Laboratory, stock number 32033‐JAX) with animals expressing Cre recombinase under the choline acetyltransferase promoter (Jackson Laboratory, stock number, 006410). Tissue‐specific knockout mice were created by breeding animals with loxP sites flanking exon 4 of mouse sirtuin 1 (Jackson Laboratory, stock number 029603) with animals expressing Cre recombinase under the choline acetyltransferase promoter (Jackson Laboratory, stock number 006,410) or the muscle creatine kinase promoter (Jackson Laboratory, stock number 006475). High copy SOD1^G93A^ animals (Jackson Laboratory, stock number 004435) were maintained on a C57Bl6 background and monitored for copy number drops using quantitative PCR genotyping.

Survival analysis was performed by breeding SOD1^G93A^ animals with SIRT1 motor neuron transgenic animals to identify SOD1^G93A^ offspring with litter‐ and gender‐matched SIRT1 MNTg SOD1^G93A^ siblings. ALS mice were monitored, and animals with nondisease‐related deaths were excluded from analysis along with their sib pairs. Endpoint was defined as the day when animals became unable to right themselves within 15–30 s when placed on either side. Statistical analysis was performed with log‐rank (Mantel–Cox) testing, and Kaplan–Meier curves were generated using GraphPad Prism 5 software.

### 4.2 Western blot

Spinal cords and gastrocnemius muscles were dissected from three male SOD1^G93A^ mice and gender‐ and litter‐matched control mice at 5.5 months and stored at −80°C until processed for western blot analysis. Tissues were homogenized in RIPA buffer with protease and phosphatase inhibitors (Roche) and centrifuged for 10 min at >10,000× *g*. Supernatant proteins were normalized using BCA assay (Pierce) and subjected to SDS–PAGE using the Criterion gel system (Bio‐Rad) followed by transfer to PVDF membranes. Standard western blot procedures were followed using 5% milk in PBS as blocking solution and antibodies to SIRT1 (Sigma, catalog # HPA 006295) and GAPDH (Sigma, catalog # G9545) at 1:1,000 dilution. HRP‐labeled secondary antibodies were used at 1/5,000 dilution, and membranes were developed with SuperSignal (Pierce) or Clarity (Bio‐Rad) for ECL detection.

### 4.3 RNA analysis

RNA extraction was performed from whole spinal cords on eight male mice at 3–4 months and eight male mice at 18–24 months to examine aging‐related changes. Whole spinal cords from three SOD1^G93A^ mice (two female and one male) and three wild‐type control mice (two female and one male) at 5 months were used to examine changes due to ALS. Analysis of three SOD1^G93A^ mice (two female and one male) and three MNTg‐SOD1^G93A^ mice (two female and one male) at 5 months was used to examine changes due to SIRT1 overexpression in the SOD1^G93A^ model. RNA was extracted with TRIzol and purified using RNA Miniprep kit (Qiagen). RNA libraries were sequenced on the Illumina HiSeq 2000 platform.

RNA‐seq data were aligned and summarized using STAR version 2.5.3a, RSEM version 1.3.0 (Li & Dewey, 2011), samtools/1.3 (Li et al., 2009), and the ensemble version 88 annotation of the mm10 mouse genome assembly. Differential expression analysis was done with R version 3.4.0 and DESeq2 version 1.16.1 (Anders & Huber, 2010), and differentially expressed genes were defined as those having an absolute log2 fold change greater than 1 and an adjusted p‐value less than 0.05. Data parsing and clustering were done using Tibco Spotfire Analyst 7.6.1. Mouse genes were mapped to human orthologs using Mouse Genome Informatics (https://www.informatics.jax.org/) orthology report, and GSEA analysis was performed using javaGSEA version 2.3.0 with msigDb version 6.0 (Subramanian, Tamayo, & Mootha, 2005) gene sets. The 404 genes increased both in the ALS vs. wild‐type and in the old vs. young comparisons were filtered to select those with at least 25 counts in all replicates in order to select transcripts with the most consistent and high level of expression in our dataset. The ImmGen data browser (https://rstats.immgen.org/MyGeneSet/) was used to compare this dataset with gene signatures characteristic of different immune cell populations. The data described in this study are available under the GEO accession number GSE106803.

### 4.4 Immunohistochemistry

Animals were euthanized, and transcardial perfusion was performed with cold PBS followed by fresh 4% paraformaldehyde (PFA) in PBS, followed by an additional 30 min of postfixation in 4% PFA. The extensor digitorum longus (EDL) muscle was dissected and incubated for 1 hr at room temperature in blocking solution (0.5% Triton X‐100, 3% BSA, 5% goat serum in PBS). EDL muscles were then incubated with Alexa‐555‐conjugated α‐bungarotoxin (fBTX, Life Technologies; 1:1,000) and synaptotagmin‐2 (znp‐1, Zebrafish International Resource Center; 1:100) for 24 hr at 4°C in blocking solution. Following three washes with PBS, EDL muscles were incubated for 2 hr with Alexa‐488‐conjugated secondary anti‐mouse IgG2a antibody (Life Technologies; 1:1,000) diluted in blocking buffer. EDL muscles were whole‐mounted onto slides using Vectashield (Vector Labs) for confocal analysis.

### 4.5 Neuromuscular junction analysis

To analyze structural features at NMJs, maximum intensity projections of confocal stacks were created using ZEN software (Zeiss). The presynaptic region of motor axons was visualized with an antibody against synaptotagmin‐2 (Syt2), a protein that associates with synaptic vesicles, and the postsynaptic region was marked with fluorescently tagged alpha‐bungarotoxin, which binds with high affinity to muscle nicotinic acetylcholine receptors (AChRs). Fully innervated NMJs were characterized by the nearly perfectly apposition between the presynaptic and postsynaptic regions. NMJs were scored as partially innervated if there was incomplete overlap between pre‐ and postsynaptic regions. Denervated NMJs were characterized by nerve endings that were completely missing from postsynaptic sites.

### 4.6 Statistical analysis

Densitometry was performed using ImageJ software, and statistical analysis was carried out using GraphPad Prism 5 software. Unpaired *t* tests were used to compare levels of SIRT1 normalized to GAPDH in wild‐type and G93A animals for each tissue analyzed. NMJ analysis was performed by imaging over 100 NMJs per mouse in 10–12 different regions of the EDL muscles. Unpaired *t* tests were used to compare NMJs of wild‐type mice with animals having altered levels of SIRT1 from the same experimental cohort with litter‐ and gender‐matched sib pairs as described in prior studies (Valdez et al., 2010, 2012 ). Survival curve analysis was performed using log‐rank (Mantel–Cox) testing and Kaplan–Meier curves. Values of *p* < 0.05 were considered to be significant. The results are shown as mean ± *SD*.

## AUTHOR CONTRIBUTIONS

A.H. and L.G. involved in conceptualization. A.H., G.V., and C.W. performed formal analysis. T.H. N.M., A.H., G.V. K.P., and C.W. performed investigation. G.V., A.H., C.W., T.H., N.M., K.P. and L.G. involved in writing, review, and editing. A.H. and L.G. performed funding acquisition.
