# Supplementary figures and images for: SIRT1 deacetylase in aging‐induced neuromuscular degeneration and amyotrophic lateral sclerosis
Source: Aging Cell. 2018 Oct 8;17(6):e12839. doi: 10.1111/acel.12839 (PMC6260920; doi:10.1111/acel.12839)

**A**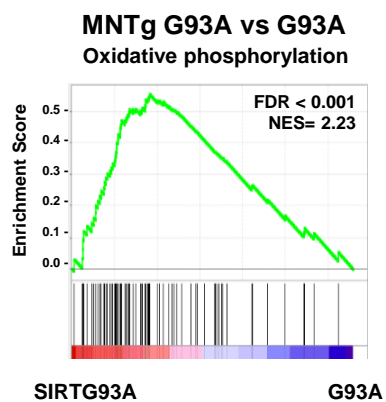**B**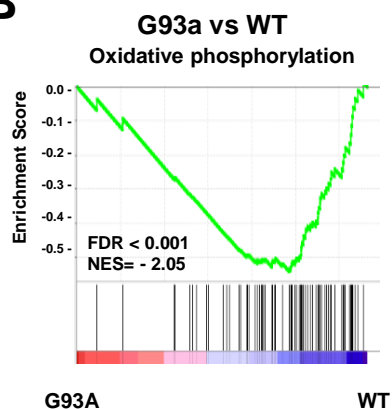**C**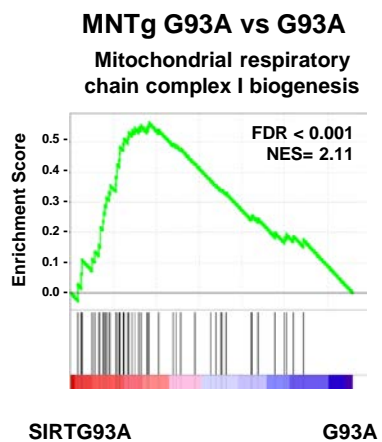**D**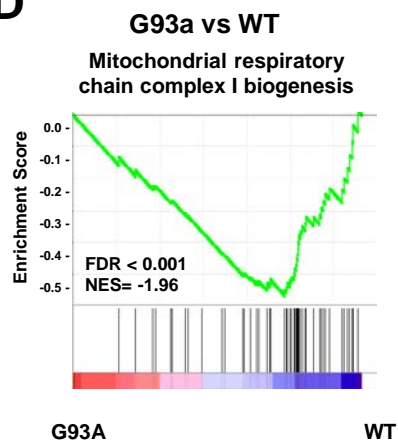

Supplement: Supplementary file 2 [file ACEL-17-e12839-s002.pdf]
