# Supplementary material for: SIRT1 deacetylase in aging‐induced neuromuscular degeneration and amyotrophic lateral sclerosis
Source: Aging Cell. 2018 Oct 8;17(6):e12839. doi: 10.1111/acel.12839 (PMC6260920; doi:10.1111/acel.12839)
Supplement: Supplementary file 3 [file ACEL-17-e12839-s003.pdf]

A

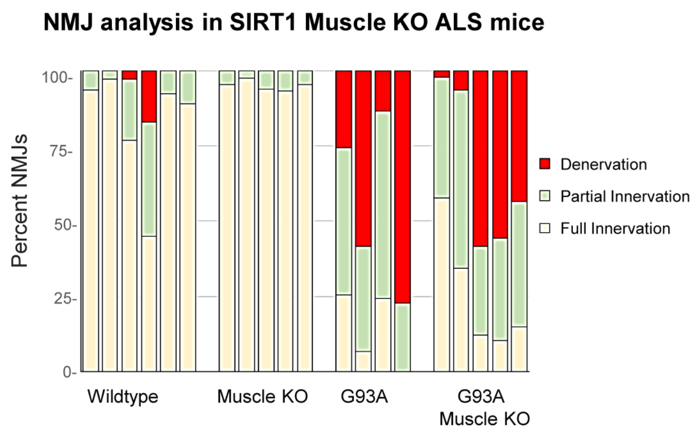

NMJ analysis in SIRT1 Muscle KO ALS mice
Percent NMJs
100-
75-
50-
25-
0-
Denervation
Partial Innervation
Full Innervation
Wildtype
Muscle KO
G93A
G93A
Muscle KO

B

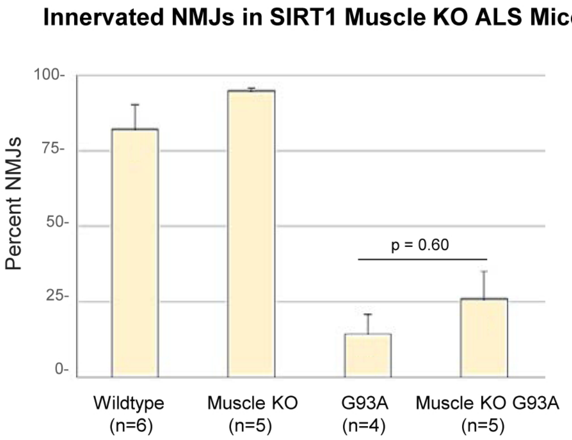

Innervated NMJs in SIRT1 Muscle KO ALS Mice
Percent NMJs
100-
75-
50-
25-
0-
p = 0.60
Wildtype
(n=6)
Muscle KO
(n=5)
G93A
(n=4)
Muscle KO G93A
(n=5)

C

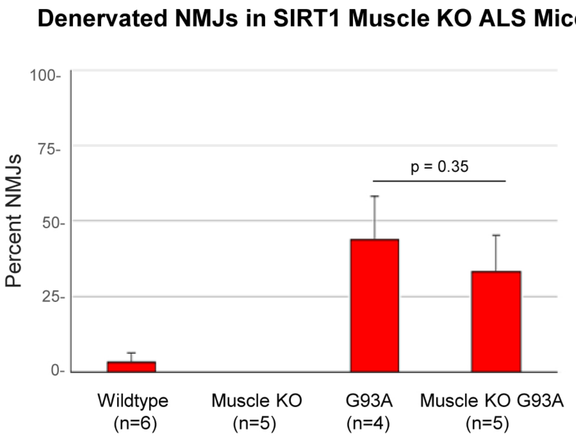

Denervated NMJs in SIRT1 Muscle KO ALS Mice
Percent NMJs
100-
75-
50-
25-
0-
p = 0.35
Wildtype
(n=6)
Muscle KO
(n=5)
G93A
(n=4)
Muscle KO G93A
(n=5)
