# Supplementary material for: SIRT1 deacetylase in aging‐induced neuromuscular degeneration and amyotrophic lateral sclerosis
Source: Aging Cell. 2018 Oct 8;17(6):e12839. doi: 10.1111/acel.12839 (PMC6260920; doi:10.1111/acel.12839)
Supplement: Supplementary file 1 [file ACEL-17-e12839-s001.pdf]

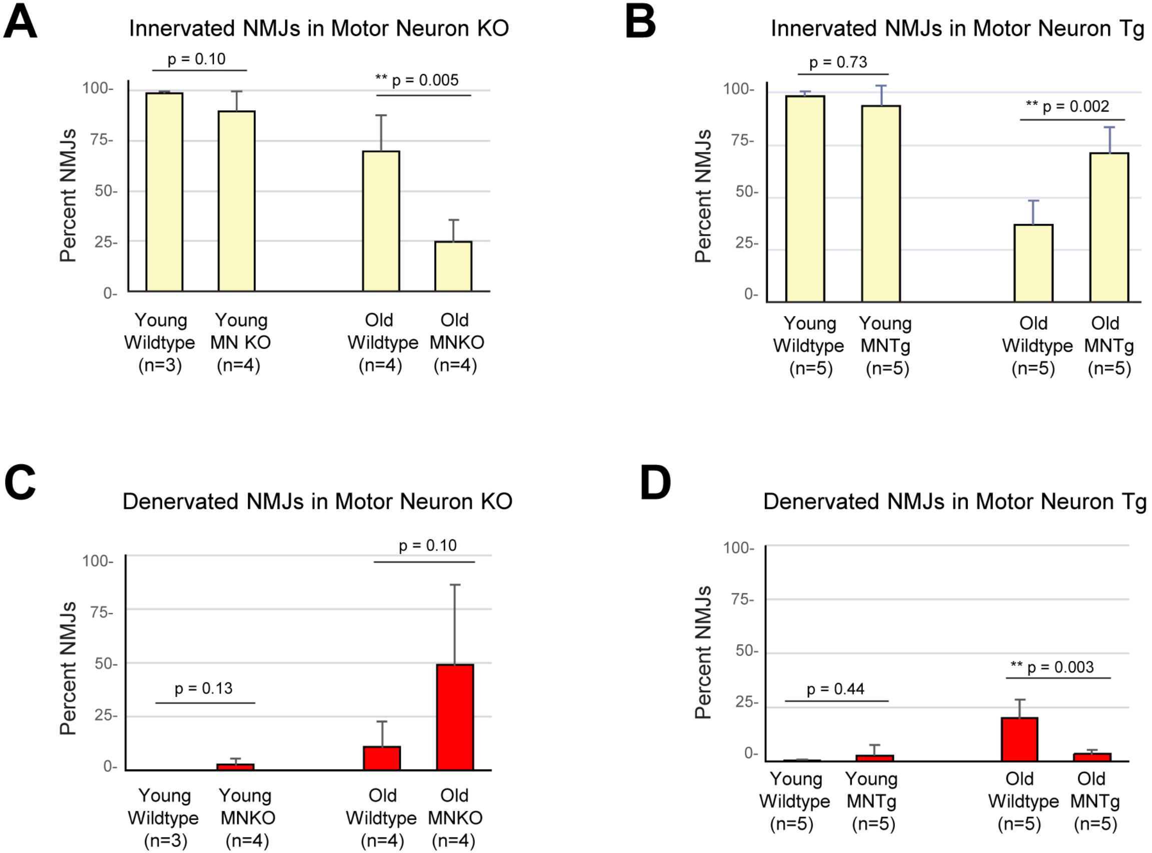
A
Innervated NMJs in Motor Neuron KO
p = 0.10
** p = 0.005
Percent NMJs
100-
75-
50-
25-
0-
Young Wildtype (n=3)
Young MN KO (n=4)
Old Wildtype (n=4)
Old MNKO (n=4)
B
Innervated NMJs in Motor Neuron Tg
p = 0.73
** p = 0.002
Percent NMJs
100-
75-
50-
25-
0-
Young Wildtype (n=5)
Young MNTg (n=5)
Old Wildtype (n=5)
Old MNTg (n=5)
C
Denervated NMJs in Motor Neuron KO
p = 0.10
p = 0.13
Percent NMJs
100-
75-
50-
25-
0-
Young Wildtype (n=3)
Young MNKO (n=4)
Old Wildtype (n=4)
Old MNKO (n=4)
D
Denervated NMJs in Motor Neuron Tg
p = 0.44
** p = 0.003
Percent NMJs
100-
75-
50-
25-
0-
Young Wildtype (n=5)
Young MNTg (n=5)
Old Wildtype (n=5)
Old MNTg (n=5)
